# Grain Intake and Human Health

**DOI:** 10.3390/nu12123733

**Published:** 2020-12-04

**Authors:** Fabiana Zingone

**Affiliations:** Department of Surgery, Oncology and Gastroenterology, Gastroenterology Section, University Hospital of Padua, 35128 Padua, Italy; fabiana.zingone@unipd.it

Wheat is one of the most consumed cereal grains worldwide and represents an important part of the human diet. Together with maize and rice, wheat species account for over 70% of the total cereal production worldwide, being of great nutritional and economic importance. The protein content of wheat is between 7% and 22%, with gluten constituting about 80% of the total protein of the seed [1]. In recent years, several clinical conditions have been related to gluten and more generally to wheat intake. This Special Issue on “Grain Intake and Human Health” presents original research communications and comprehensive reviews on topics of broad interest to researchers, offering a robust and critical updated view on the relationship between grain intake and human health, with a particular focus on gluten-related diseases. These latter include three broad categories: immune-mediated disorders including celiac disease, dermatitis herpetiformis, and gluten ataxia; allergic reactions, such as wheat allergy; and non-celiac gluten sensitivity, characterized by self-reported symptoms improving with a gluten-free diet in subjects for whom other major gluten-related disorders have been excluded.

Schiepatti A et al. [2] in their review help the readers to identify diagnostic errors, classified into missed, delayed, or wrong diagnoses which may result in overtreatment of patients wrongly started on a gluten-free diet, or in severe diagnostic delays impacting on long-term morbidity and mortality and resulting in unnecessary spending of health-care resources. Mainly, the authors underline the need for compliance with international guidelines and adoption of a methodological diagnostic approach while the patient is on a normal gluten-containing diet.

The mainstay treatment of these disorders is based on the exclusion of gluten from the diet, and subjects should be educated to avoid any foods derived from wheat, barley, or rye. However, the gluten-free diet is often difficult to maintain in our societies or during travels, therefore it can impact the patients’ quality of life. 

Marsilio et al. [3] analyzed this aspect, evaluating which factors can influence the quality of life of adult celiac patients during follow-up. The authors collected data on 100 patients on a gluten-free diet, finding an overall high quality of life. However, the “health concerns” subscale score was significantly lower in subjects aged more than 35 years compared to younger subjects and the quality of life in gluten-free diet -adherent patients tended to be higher compared to subjects who were non-compliant, with a significantly higher percentage of patients with a low score for the “dysphoria” subscale. 

The same group from Padua (Italy) [4] investigated the level of nutritional knowledge in a cohort of 96 celiac patients on a gluten-free diet in comparison with 96 patients with inflammatory bowel disease (IBD) in clinical remission and 65 healthy subjects, using the Moynihan validated questionnaire to measure the nutritional knowledge. The authors found that that celiac patients were less aware of nutritional recommendations compared with healthy subjects, and were less able to identify nutrient sources compared with IBD patients and to choose healthy food compared with both groups. Therefore, the authors concluded that celiac patients tend to focus their diet on gluten avoidance, while IBD patients tend to follow a healthier diet, probably because they believe that diet plays a major role in regulating inflammation and, therefore, their symptoms and that a dietitian consultation at celiac disease diagnosis is recommended. 

Besides the difficulties related to the gluten-free diet, some patients have persistent symptoms despite diet adherence and this may be due to a high sensitivity to traces of gluten. Moreover, patients can struggle in achieving full restoration of the gut microbiota, which plays a role in nutritive compounds processing, and absorption. Therefore, we included in this Special Issue two reviews on possible safe pharmacological treatments complementing the gluten-free diet. Wei et al. [5] focused their review on oral enzyme therapy, employing gluten-degrading enzymes, discussing their origin and activities, their clinical evaluation, and challenges for therapeutic application. The authors pointed out the importance that such enzymes are active under gastro-duodenal conditions, quickly neutralize the T cell-activating gluten peptides, and are safe for human consumption, and that they must cleave the otherwise unusual glutamine and proline-rich domains characteristic of antigenic gluten peptides. Marasco et al. [6] instead focalized their review on the supplementation of the gluten-free diet with probiotics, such as Bifidobacterium and Lactobacilli, describing the results of pivotal studies which reported the potential to restore gut microbiota composition and to pre-digest gluten in the intestinal lumen, reducing the inflammation associated with gluten intake, the intestinal permeability, and the cytokine and antibody production. The authors also reported some data on the inclusion of prebiotics in the gluten-free diet which have the capacity to stimulate the growth of potentially health-promoting bacteria strains. However, the authors concluded that the evidence is still insufficient to justify their use in clinical practice. 

In this Special Issue, we also took into account possible complications related to celiac disease, such as a low bone mineral density and high fracture risk, and the possible role on these conditions of the gluten-free diet alone, without any vitamin supplementation. In particular, Ciacci et al. [7] investigated the level of 25-hydroxy-vitamin D [25(OH)D], 1,25-dihydroxy-vitamin D [1,25(OH)2D], and related analytes in celiac disease patients to evaluate their relationships to peripheral BMD as assessed by peripheral quantitative computed tomography. The authors concluded that adult celiac patients at diagnosis compared to those on the gluten-free diet had lower 25(OH)D, higher PTH, and higher 1,25(OH)2D in the absence of a difference in serum calcium and phosphorus. 25(OH)D and 1,25(OH)2D, even below the normal range, were not associated with BMD, and therefore the authors do not support the use of vitamin D supplementation for all celiac adults.

Since the importance given to the whole grain in recent times, we also included a paper on a condition not included in the category of gluten-related disorders, and in whom grain intake could report a benefit. In particular, Kashino et al. [8] examined the prospective association between whole grain consumption and the development of hypertension in Japan. The study included 944 working Japanese adults aged 19–68 years who had no hypertension at baseline and completed a three-year follow-up survey. Whole grain consumption was assessed via a self-administered dietary questionnaire. After three years, 9.4% (86 cases) of the study participants had developed hypertension. More frequent whole grain consumption, classified as an intake frequency of “sometimes or always”, was associated with lower odds of hypertension compared with no consumption. 

Finally, another aspect of the possible role of the grain in human health can be seen through a low-FODMAP diet (i.e., a low oligosaccharide, disaccharide, monosaccharide, and polyol diet) which is associated with an improvement in gastrointestinal symptoms and is characterized by the elimination of wheat, barley, spelt, rye, and all the other gluten-containing cereals, for a limited period of time. Gravina et al. [9] published, in this issue, a real-life evaluation on the adherence and effect derived from the FODMAP diet on irritable bowel disease, which is one of the most important socio-economic health problems, whose etiopathogenesis is not completely known. The authors enrolled 120 patients who underwent a low-FODMAP diet for six weeks, followed by a gradual weekly reintroduction of every category of food for three months. The authors reported a good patient adherence to the diet and a statistically significant decrease in abdominal pain, bloating, flatulence, diarrhea, constipation, and neurological bowel dysfunction score at the end of the diet. These results remained constant in the follow-up period, recommending the use of a low-FODMAP diet regimen in patients with irritable bowel syndrome in order to control the symptoms and improve the quality of life.
